# Broad-range and effective detection of human noroviruses by colloidal gold immunochromatographic assay based on the shell domain of the major capsid protein

**DOI:** 10.1186/s12866-020-02084-z

**Published:** 2021-01-11

**Authors:** Meng Xu, Feifeng Lu, Chenang Lyu, Qingping Wu, Jumei Zhang, Peng Tian, Liang Xue, Ting Xu, Dapeng Wang

**Affiliations:** 1 Department of Food Science and Technology, School of Agriculture and Biology, Shanghai Jiao Tong University, Shanghai, 200240 China; 2 State Key Laboratory of Applied Microbiology Southern China, Guangdong Provincial Key Laboratory of Microbial Culture Collection and Application, Guangdong Open Laboratory of Applied Microbiology, Guangdong Institute of Microbiology, Guangzhou, 510070 China; 3 Western Regional Research Center, Agricultural Research Service-United States Department of Agriculture, Produce Safety and Microbiology Research Unit, Albany, CA 94706 USA

**Keywords:** Human norovirus, Broad-range detection, S domain of VP1, Colloid gold, Immunochromatographic assays

## Abstract

**Background:**

Human noroviruses (HuNoVs) are a major cause of nonbacterial gastroenteritis in all age groups worldwide. HuNoVs can be detected in vitro using molecular assays such as RT-PCR and RT-qPCR. However, these molecular-based techniques require special equipment, unique reagents, experienced personnel, and extended time to obtain results. Besides, the diversity of viral genotypes is high. Therefore, methods that are rapid, broad-range and effective in the detection of HuNoVs are desiderated for screening the feces or vomit of infected people during outbreaks.

**Results:**

In this study, a colloidal-gold-based immunochromatographic assay (ICA) was developed for effective detection of HuNoVs in clinical samples. Monoclonal antibodies (MAbs) against the shell (S) domain in the major capsid protein of HuNoVs were used in the ICA. The limitations of detection for HuNoVs in clinical samples were 1.2 × 10^6^ genomic copies per gram of stool sample (gc/g) and 4.4 × 10^5^ gc/g for genogroup I and II (GI and GII) HuNoVs, respectively. A total of 122 clinical samples were tested for HuNoVs by ICA and compared against RT-qPCR. The relative sensitivity, specificity and agreement of ICA was 84.2% (95% CI: 83.6–84.8%), 100.0% (95% CI: 98.5–100.0%) and 87.7% (95% CI: 85.6–89.8%), respectively. No cross-reaction with other common enteric viruses or bacteria was observed. The ICA detected a broad range of genotypes, including GI.1, GI.3, GI.4, GI.6, GI.14, GII.2, GII.3, GII.4, GII.6, GII.13, and GII.17 HuNoVs.

**Conclusions:**

This study demonstrates that ICA targeting the S domain of VP1 is a promising candidate for effectively identifying the different genotypes of HuNoVs in clinical samples with high sensitivity and specificity.

**Supplementary Information:**

The online version contains supplementary material available at 10.1186/s12866-020-02084-z.

## Background

Human noroviruses (HuNoVs) are single-stranded RNA, non-enveloped viruses in the *Caliciviridae* family. The genome has three open reading frames (ORFs, 1 through 3), where ORF2 encodes a major capsid protein referred to as VP1. VP1 consists of a shell (S) domain and a protruding (P) domain. The S domain is the most highly conserved region in VP1, and forms a shell surrounding the RNA genome, while the P domain of VP1 contains the most variable sequence [1–3]. HuNoVs are divided into 5 genogroups based on the VP1, including genogroup I (GI), II (GII), IV (GIV), VIII (GVIII), and IX (GIX) [4]. GI and GII HuNoVs are the major epidemic strains circulating worldwide [5].

HuNoVs are the most common causes of epidemic gastroenteritis worldwide [6–8]. The HuNoVs infections cause about 700 million illnesses and over 200 thousand deaths globally every year [9, 10]. HuNoVs are highly-infectious, and infected patients can shed high titers of virus particles with strong resistance to environmental factors [11, 12]. The establishment of an efficient in vitro cell culture for HuNoVs remains challenging [13], therefore, the detection of the virus mainly depends on molecular approaches such as RT-PCR and RT-qPCR [14, 15]. These molecular approaches require specialized equipment, unique reagents, and significant time for sample preparation and assay execution, which make them impractical for either clinical use or on-site assays of field samples requiring quick results. To efficiently control the spreading of HuNoVs in time, a highly effective, safe, and portable point-of-care testing is of great importance in monitoring and managing the spread of an outbreak.

**Fig. 1:**
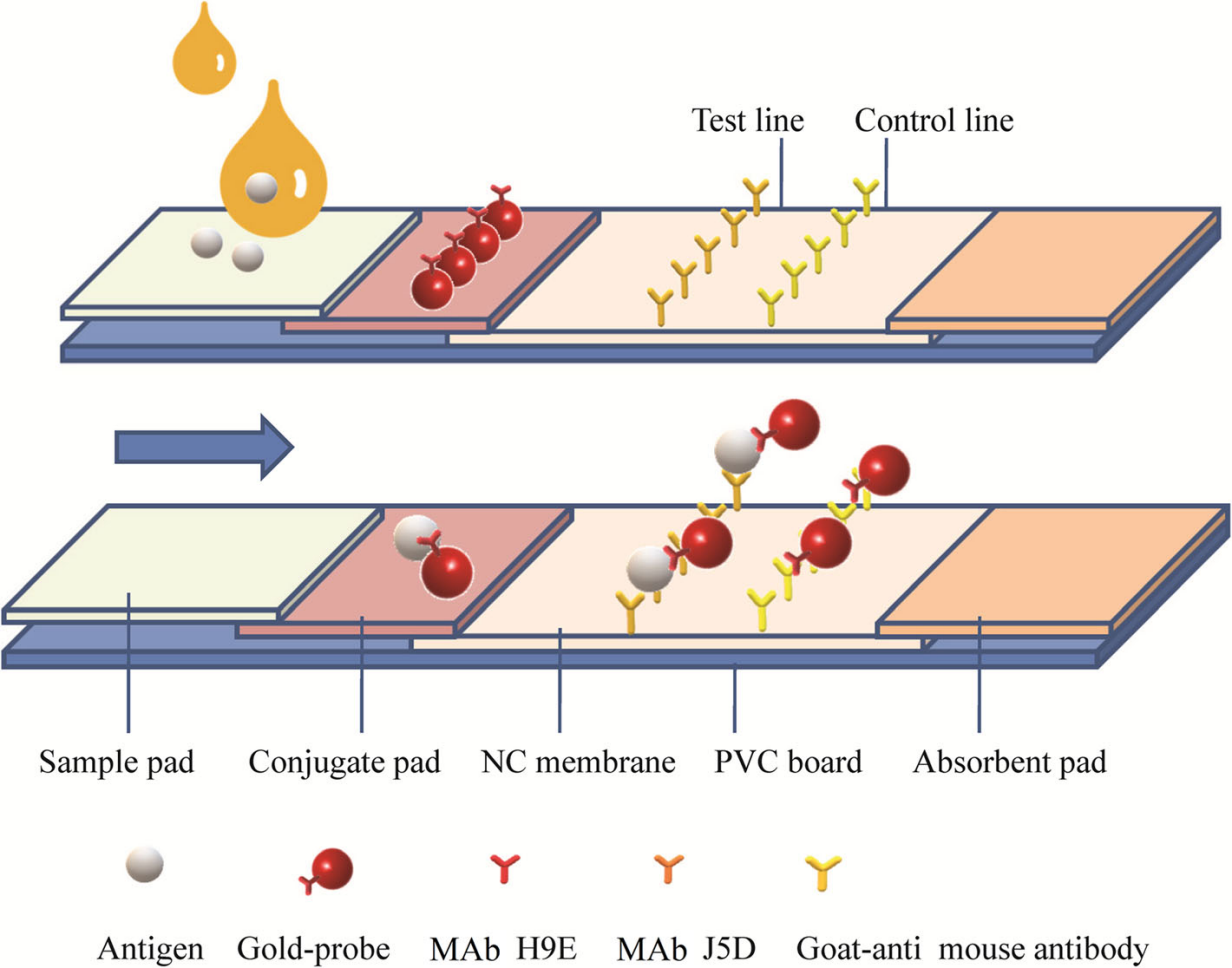
Schematic diagram of ICA test. MAb H9E was used as labeled antibody with colloidal gold particles (in red); MAb J5D was used as capture-antibody in the T line; goat-anti mouse MAb Ig G was used in the C line. Arrow indicates the direction of the movement of antigens (capsid protein of noroviruses)

An immunochromatography assay (ICA) determines the presence or absence of a target analyte, such as pathogens or biomarkers [16, 17]. A schematic diagram of ICA is shown in Fig. 1. In an ICA test, an antibody against the viral capsid is labeled with gold particles. The labeled antibody captures the viral capsid and another antibody coated on the solid-phase carrier, thus the virus-antibody-colloidal gold particle combinations cause aggregation to occur and cause a color change to red indicating positive results. ICAs do not require any specialized equipment and minimal training is needed to perform the test. Results can be visually displayed within a few minutes. Currently, several ICAs used for the detection of bacterial and viral pathogens have been reported [18, 19]. However, the limitations of the ICA kits in HuNoVs detection were obvious, such as the specific detection of genotypes and low sensitivity [20]. Therefore, a broad-range and highly effective ICA is needed for point-of-care testing of HuNoVs.

Li et al. reported that a MAb against the S domain of VP1 could cross-react with GI, GII, GIII, and GV of noroviruses (NoVs) [21]. Yoda et al. reported that two MAbs generated against the S domain of GII HuNoVs capsid protein also recognized the viral protein of GI HuNoVs [22]. Besides, Parra et al. identified a broad cross-reactive epitope in the S domain of the NoVs capsid [2]. Therefore, the S domain of VP1 is highly conserved and serves as a good candidate for detecting multiple genotypes of HuNoVs. However, few immunoassays have been reported to detect HuNoVs targeting the S domain of VP1. In this study, three MAbs were generated against the S domain of VP1 from GII.4 HuNoV and used to develop an ICA for broad and effective detection of both GI and GII HuNoVs.

## Results

### Selection of MAbs recognizing different epitopes on the S domain of VP1 for ICA

Selection of MAbs recognizing different epitopes on S domain of VP1 could enhance the sensitivity and specificity of the assay. Three MAbs were selected and named H9E, B4H, and J5D, further abbreviated as H, B, and J, respectively. The values of the displacement factor (*I*) of B-H, H-B, B-J, J-B, H-J, and J-H combinations determined by ELISA were 32.8, 28.9, 34.1, 29.6, 31.2, and 22.7%, respectively. *I* value greater than 10.0% indicated that two MAbs recognized different epitopes on the S domain of VP1. Among the *I* values of the six pairs, B-J showed the best matching effect. The results were further tested by the ICA assay to find the best combination of MAbs binding to the gold particles (Table 1). Finally, H was selected as the antibody for the colloid labeled gold, and J as the test-line capture antibody, which is shown in both the test and control lines.

**Table 1:** Results obtained from different combinations of the three antibodies on the colloidal gold platform

	Colloidal-gold MAb	Test line MAb	Test line	Control line
BH:	B4H	H9E	+	+
HB:	H9E	B4H	++	+
BJ:	B4H	J5D	+	++
JB:	J5D	B4H	++	+
**HJ:**	**H9E**	**J5D**	**++**	**++**
JH:	J5D	H9E	+	+

“+” represented the color depth. The score was evaluated by multiple operators with hidden labels

### Pretreatment methods to expose the S domain of VP1 in the clinical samples

Unlike the P domain of VP1, the S domain of VP1 is hidden inside of the viral capsid. Two methods were used to expose the S domain of VP1 in clinical samples and to inactivate the virus. By increasing the temperature from 60 °C to 80 °C, the OD readings gradually increased (roughly from 0.2 to 1.0). However, the OD readings dramatically decreased when the temperature was greater than 90 °C (less than 0.2). A 3-min treatment at 80 °C obtained the best heat-treatment results (Fig. S1, see Additional file 1). A progressive improvement in detection was displayed when pH increased from 7.0 to 9.0 (optimal at pH = 9.0, consistent with the best effect of heat treatment) (Fig. S2, see Additional file 1). Alkali treatment performed better than heat-treatment, the OD readings were easy to control and stable over the time tested, and there was no significant difference between the best 10-min and the worst 5-min treatment (*p* = 0.131 > 0.05). For simplicity and repeatability of the experiment, alkali treatment (samples were treated to pH = 9.0 at room temperature for 10 min) was used to expose the S domain of VP1 in all clinical samples before ICA testing.

### The sensitivity and specificity of ICA

**Fig. 2:**
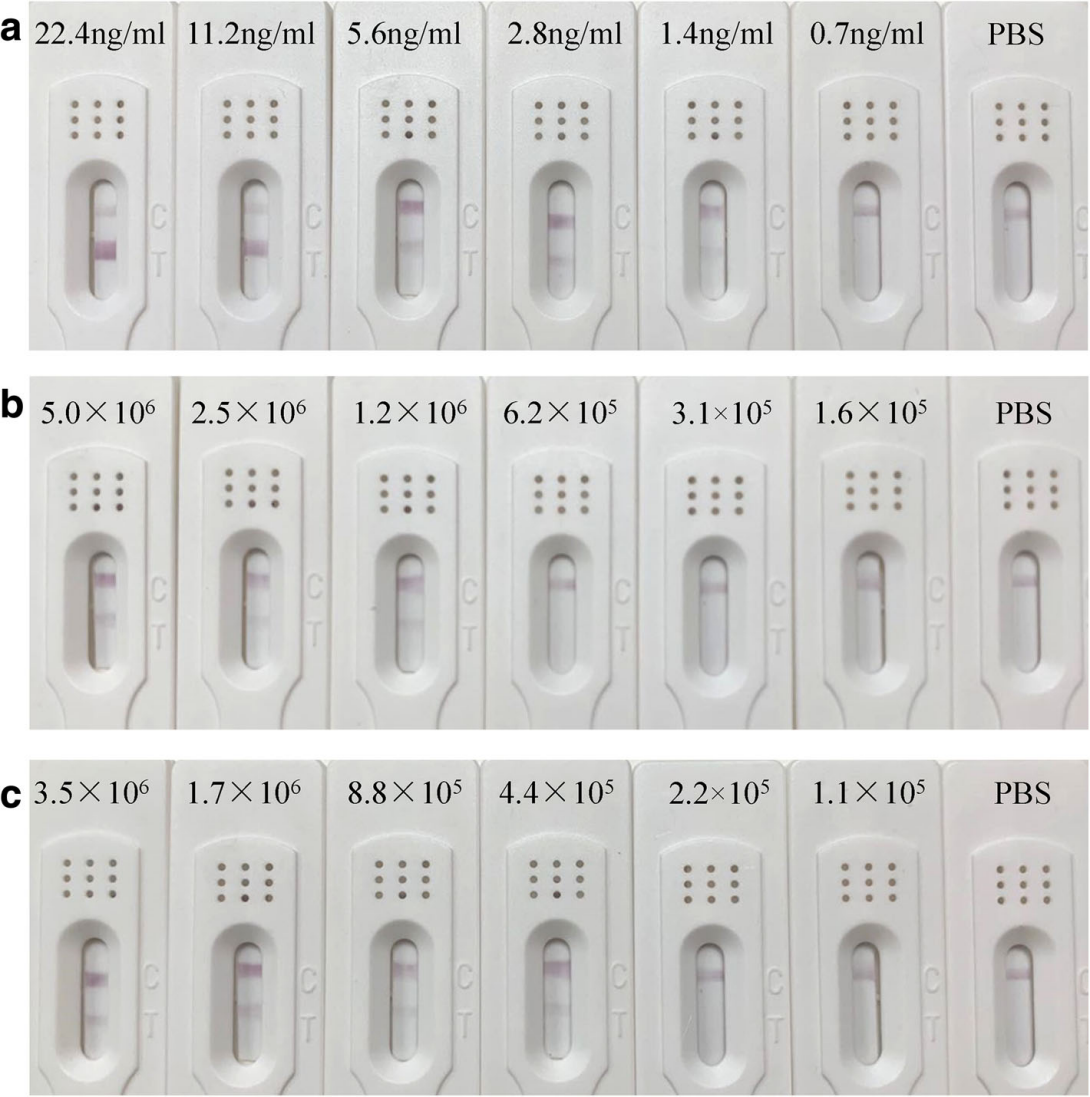
LOD of ICA for purified S domain of VP1 and clinical samples. **a** Sensitivity for S domain of VP1 (two-fold dilutions from 22.4 ng/ml to 0.7 ng/ml), **b** Sample 57,404 (GI.1) (two-fold dilutions from 5.0 × 10^6^ to 1.6 × 10^5^ gc/g) and (**c**) Sample 1717 (GII.4) of different virus copies (two-fold dilutions from 3.5 × 10^6^ to 1.1 × 10^5^ gc/g) were detected with test strips. PBS buffer (pH 7.4) was used as blank control

The sensitivity of ICA is based on the limit of detection (LOD). The LOD of ICA is determined by applying serially diluted HuNoVs on an ICA strip. The results were judged by visualization of both the test line and control line. The C_*t*_ data and the copies were recorded as shown in Table S1 (Additional file 2). The visual LOD of the ICA for the determination of the S domain of VP1 was 1.4 ng/ml. The LODs of viral genomic copies of samples 57,404 (GI) and 1717 (GII) were 1.2 × 10^6^ gc/g and 4.4 × 10^5^ gc/g, respectively. The sensitivity test was performed in triplicate, and representative results are shown in Fig. 2.

**Fig. 3:**
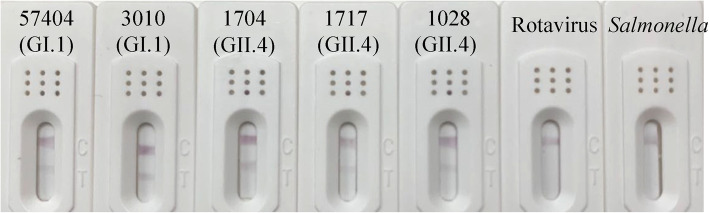
Specificity of ICA. Five HuNoVs clinical samples, Rotavirus, and *Salmonella* cultured samples were tested with the strips

The specificity of ICA was determined using a set of clinical samples with HuNoVs and other pathogens, and compared with RT-qPCR results. When the clinical samples were detected by ICA, only samples containing HuNoVs showed positive results. Clinical samples containing other enteric viruses (4 Rotaviruses, 3 Sapoviruses, 2 Astroviruses, and 4 Adenoviruses) or bacteria causing gastroenteritis (3 *Salmonellas*) all tested negative. Parts of the results are shown in Fig. 3. A comparison of the ICA and RT-qPCR results are shown in Table 2. Eighty samples were found to be positive by ICA from 95 RT-qPCR HuNoVs positive samples. The sensitivity was 84.2% (95% CI: 83.6–84.8%) (80/95). There were no false-positive results, indicating a specificity of 100.0% (95% CI: 98.5–100.0%) (27/27). The overall agreement of ICA and RT-qPCR was 87.7% (95% CI: 85.6–89.8%) (107/122). Besides, the titers of HuNoVs in 15 negative samples (1 GI.6, 1 GII.3, 12 GII.4, and 1 GII.6) are shown in Table S2 (Additional file 2). The viral loads of 15 ICA false-negative samples were below the LOD of ICA.

**Table 2:** A comparison of HuNoVs detection in stool samples between the ICA and the RT-qPCR

		ICA		
		Positive	Negative	Total
RT-qPCR	Positive	80	15	95
	Negative	0	27	27
	Total	80	42	122

Sequencing results signaled that GII.4 was the dominant genotype (72/95) followed by GI.1 (4/95) in RT-qPCR HuNoV-positive samples. Other genotypes were GI.3 (2/95), GI.4 (3/95), GI.6 (2/95), GI.14 (1/95), GII.2 (2/95), GII.3 (3/95), GII.6 (3/95) GII.13 (1/95), and GII.17 (2/95) (Table 3). ICA detected both GI and GII HuNoVs, including GI.1, GI.3, GI.4, GI.6, GI.14, GII.2, GII.3, GII.4, GII.6, GII.13, and GII.17 genotypes. Our ICA could detect all 11 genotypes. The 15 false-negative samples were because of the low titer.

**Table 3:** Detection results of HuNoVs genotypes by ICA

HuNoVs genotypes	Positive number in RT-qPCR	Number in ICA		Sensitivity (95 % CI)
		Positive	Negative	
GI.1	4	4	0	
GI.3	2	2	0	
GI.4	3	3	0	
GI.6	2	1	1	
GI.14	1	1	0	
**(GI total)**	**(12)**	**(11)**	**(1)**	**91.7 % (88.6-94.8)**
GII.2	2	2	0	
GII.3	3	2	1	
GII.4	72	60	12	
GII.6	3	2	1	
GII.13	1	1	0	
GII.17	2	2	0	
**(GII total)**	**(83)**	**(69)**	**(14)**	**83.1 % (80.0-86.3)**
**Total**	**95**	**80**	**15**	

### Stability of ICA strips

The findings demonstrated that the acceptable activity (90.0%) of the initial activity was retained for 21 days when the test strip was exposed to 60 °C. Therefore, we hypothesized that the shelf-life of ICA strips remains stable for more than 21 days at room temperature.

## Discussion

HuNoVs are recognized as one of the most important foodborne pathogens worldwide [23]. The burden of HuNoVs outbreaks is high and is reflected in the extensive infectivity and severe economic losses [24]. Acute gastroenteritis outbreaks associated with HuNoVs are particularly challenging to control because of their stability in the environment and efficient transmission in hospitals, hotels, schools, and homes [25–27]. Therefore, a broad and highly effective assay is needed to rapidly identify HuNoVs and provide emergency treatment during outbreaks.

Fecal specimens from patients could contain up to 10^9^ gc/g of HuNoVs [28], risky for the operators. Therefore, the inactivation of the viral particles in clinical samples is crucial before detection. To address this need, physical and chemical treatment methods were evaluated in this study. Our results indicated that the capsid was greatly disassembled following alkaline (pH 9.0 for 10 min) and heat (70.0 °C for 3 min) treatment. The alkali treatment performed better than the heat treatment in terms of results stability and simplicity. More important, the alkali environment also maintained the colloidal gold in a stable state to bind MAbs.

VP1 proteins (P plus S domains) can self-assemble to form virus-like particles (VLPs) which morphologically and antigenically resemble the viral particles [29, 30]. The protruding (P) domain of VP1 is highly immunogenic and similar to VLP [31]. The antigenicity of VLP and P particles is often strain-specific and not suitable for viral screening. On the other hand, the S domain of VP1 in HuNoVs is highly conserved [7]. Numerous studies have reported that two MAbs (1B4 and 1F6) of the S domain of VP1 against the capsid protein (NoV GII) reacted with NoV GI [22, 32]. In this study, MAbs against the S domain of VP1 were prepared to develop an ICA kit, which recognized VP1 from either GI or GII HuNoVs. Moreover, the results demonstrated that the kit had good sensitivity and specificity for the detection of both GI and GII HuNoVs (Table 3).

The epitopes on the S domain of VP1 are hidden in the inner layer of the viruses. They cannot be recognized by antibodies against the S domain of VP1 in the intact viral particles. Therefore, it is important to expose the S domain of VP1 for virus detection. In this ICA assay, the viral particles were disassembled by alkaline treatment to expose the S domain of VP1. It minimized the risk of infection and kept testers much safer than detection without inactivated samples. In addition, each viral particle contains 90 dimers of viral capsid [33]. Theoretically, more copies of S domain of VP1 are exposed to the MAbs after treatment, thus increasing the sensitivity. Since limited research exists on the antigenicity of the S domain of VP1, it will be meaningful to identify the epitopes of S domain in later studies.

The current molecular assays require special equipment, reagents, and experienced personnel. A simple approach with minimal hands-on time is more applicable and requisite as a screening assay. There are several available commercial immunoassays for HuNoV detection, such as Quick Ex-Norovirus, RIDA® QUICK (N1402), Immuno Search NV kit, NOROTOP+, and SD Bioline Norovirus [34–40]. However, these methods were more effective for single genotype detection. To detect multiple strains of HuNoVs, a couple of MAbs are selected for cross-reactions with various genotypes. A simple approach is urgently needed to detect a broad-range of HuNoVs.

Numerous studies report the use of IAC in the detection of NoVs. IP-NoV kit and the kit developed by Takanashi were mainly used for GII.3 and GII.4 HuNoVs detection [41–43]. Immuno Search NV kit was developed for several genotypes (GI.1, GI.11, GII.2, GII.3, GII.4, GII.5, and GII.6 VLPs), but the limit of detection was not shown [29]. SD Bioline Norovirus could detect GI.3 for GI but not GII.2, GII.6, and GII.16 for GII HuNoVs [31, 32]. Limited genotypes of HuNoVs are detected by most of the reported kits. It is noteworthy that our ICA could detect a much broader range of genotypes (including GI.1, GI.3, GI.4, GI.6, GI.14, GII.2, GII.3, GII.4, GII.6, GII.13 and GII.17) (Table 3).

Compared to RT-qPCR, the sensitivity, specificity and agreement of our ICA were 84.2% (95% CI: 83.6–84.8%), 100.0% (95% CI: 98.5–100.0%) and 87.7% (95% CI: 85.6–89.8%), respectively (Table 2). The overall results were competitive with other commercial kits. The sensitivity (84.2%) of our ICAs was higher than IP-NoV kit (ranged from 72.7 to 78.9%), Quick Ex-Norovirus (54.5%), RIDA® QUICK (from 68.8 to 82.5%), Immuno Search NV kit (75.4%) and NOROTOP+ (51.4%) (See Table S3, Additional file 3). Besides, Rotavirus, Sapovirus, Astrovirus, Adenovirus, and *Salmonella* are the most common non-norovirus viral and bacterial pathogens that also cause gastroenteritis in China [44]. None of the 16 specimens tested positive when evaluating specificity in this study. The detection using ICA was dependent on the visualization of both the test and control line. Future studies will seek to improve the proposed ICA assay.

The performance characteristics of the developed ICA kit were dependent on both the tested individual and the HuNoVs titer in the sample. There were several possibilities for false-negative results, including lower viral titer in the tested clinical samples and sensitivity of the MAbs selected. In our study, 15 false-negative samples identified had viral titers below the LOD. In future studies, the LOD of the developed kit should be improved using quantum dot or signal amplification systems. Nevertheless, the ICA targeting the S domain of VP1 is a promising approach for screening HuNoVs in clinical samples, especially during outbreaks.

## Conclusions

In this study, an ICA targeting the S-domain of the HuNoV VP1 for broad-range detection of HuNoVs is developed. This assay reduces the detection time and has a sensitivity of 84.2% (95% CI: 83.6–84.8%), and a specificity of 100.0% (95% CI: 98.5–100.0%), which is highly competitive compared with other immunological kits. Besides, no special equipment and reagents are required. Therefore, the proposed assay presents a promising approach for the screening of HuNoVs from clinical samples in the field.

## Methods

### Preparation of VP1s and the S domain of VP1

HuNoV GI.1 and GII.4 ORF2 gene fragments (GenBank Nos. M87661 and KM114291, respectively) were subcloned separately into the pET-28a prokaryotic expression plasmid. HuNoV GI and GII VP1 proteins were expressed from respective recombinant plasmid vectors pET28a-ORF2 GI.1 and GII.4 in *Escherichia coli* (*E. coli*) BL21 cells [45].

HuNoV GII.4 ORF2 (GenBank No. KM114291) was used as a template to amplify the nucleic acid fragment of the S domain. The upstream primer was 5′-GAATTCATGAAGATGGCGTCGAGTG-3′; the downstream primer was 5′- CTCGAGCTCAACTGTGGGTGGCAC-3′. *Eco*R I and *Xho* I restriction sites were appended to the 5′ ends of forward and reverse primers, respectively. After amplification and digestion, the nucleic acid fragment of the S domain was inserted into a pSmart vector (Frdbio, China) to generate recombinant pSmart-S. The recombinant S domain of VP1 was induced and expressed, as described in a previous study [46].

### Preparation of anti-S domain of VP1 MAbs

The MAbs against the S domain of VP1 were prepared as described in our previous study [47]. Three MAbs (H9E, B4H, and J5D, further abbreviated as H, B, and J, respectively) were selected for their ability to bind the pSmart-S expression product, but not with *E. coli* residual proteins. The three MAbs also demonstrated specificity against both GI and GII HuNoV VP1, which was confirmed by Western Blot analysis.

### Selection of MAbs recognizing different epitopes of the S domain of VP1 by ELISA assay

Each reaction of the ICA assay utilizes a labeled MAb and an immobilized (“capture”) MAb. Since the conditions for the labeled MAb (40 μg/ml) and the immobilized Mab (2.0 mg/ml) were different, all six pair permutations of the three MAbs (B-H, H-B, B-J, J-B, H-J, and J-H) were tested. First, the three MAbs (B, H, and J) were two-fold serially diluted and tested for their single epitope saturation concentrations using a recombinant S domain of VP1 (100.0 μg/well). A concentration one step lower than the concentration with a significant decrease in OD_450_ value was defined as a single epitope saturation concentration of the MAbs and named B_1_ (or H_1_, J_1_). The S domain of VP1 (100.0 μg/well) was coated overnight at 4 °C, and 100.0 μl epitope-saturated MAb (e.g., B) was added and incubated at 37 °C for 1 h. After washing, 100.0 μl of saturated MAb (e.g., H) in the test pair was incubated under similar conditions. The OD_450_ was recorded as BH_2_. The displacement factor (*I*) was defined as the ratio of the increased effect of cross-reactivity over the separate effect of the second antibody. If *I* > 10% [48], this indicated that the recognition sites of the two MAbs were distinct. *I* values was calculated using the formula *I*_(e.g., BH)_ = (BH_2_ - B_1_)/H_1_ × 100%.

### Preparation of the colloidal gold-labeled MAbs

Colloidal gold particles with a mean particle diameter of 25.0 nm were produced. One hundred ml of 0.01% (w/v) chloroauric acid (HAuCl_4_) (Aladdin, Shanghai, China) was thoroughly boiled for 3 min, and 2.0 ml of 1.0% (w/v) sodium citrate (Aladdin, Shanghai, China) quickly added into the solution with magnetic stirring for 30 min. The color gradually changed from yellow to black–blue and finally brilliant red. After stirring for a few minutes at low speed, the colloidal gold suspension could cool down and stored in the dark at room temperature. The total volume was made up to the original volume (100.0 ml) using ultrapure water. To determine the size and distribution of the gold nanoparticles, a transmission electron microscopy (Tecnai G2 spirit Biotwin, USA) was used to scan the colloidal gold solution at 120 KV. The OD value of the colloidal gold solution was measured at 400–680 nm using an ultraviolet spectrophotometer (Tecan Sunrise, Switzerland).

Both physical and chemical crosslinking methods have been used for the conjugation of gold colloids and MAbs [49]. Chemical crosslinking is more stable but the functional groups in MAb are likely to be affected. All the functional domains are retained when MAbs are conjugated by the physical method [50]. In this study, the physical method was used for conjugation [49]. The optimal pH, dose, and BSA concentration during the conjugation of gold colloids and MAbs were evaluated (Fig. S3 and Table S4–6, Additional files 4 and 5). Briefly, 200.0 μl of a MAb (i.e.H9E) was incubated with 0.5 ml of colloidal gold (pH 9.0) for 30 min at room temperature and with gentle stirring. Then, 50.0 μl BSA (Amresco, United States) at different concentrations were added into the colloidal gold as a blocking buffer to stabilize the gold-labeled antibody. After 15 min incubation, centrifugation at 8000×g at 4 °C for 20 min allowed the collection of the colloidal gold-antibody complex as a pellet, and unbound antibodies were retained in the supernatant. The presence of black massive deposits on the wall of the tube should be avoided during the centrifugal process. The conjugated colloidal gold-antibody was re-suspended in 50.0 μl of dissolution buffer (PBS, pH 9.0 containing 10.0% w/v sucrose (Sangon Biotech, Shanghai, China), 0.2% (w/v) PVA-205 (Aladdin, Shanghai, China), 0.2% (v/v) Tween-20 (Aladdin, Shanghai, China), and BSA (3.0, 2.5, 2.0, 1.5, 1.0 and 0.5%, w/v). The conjugation was confirmed by UV-vis spectroscopy using unlabeled gold particles. A 50.0 μl colloidal gold-antibody mixture was evenly dispensed on the 0.5 cm × 2.5 cm conjugated pad and dried for 3 h at room temperature.

### Selection of MAbs used on the colloidal gold platform

ICA performance was critically dependent on a combination of the optimal antibody sandwich pair with colloidal gold. ELISA results were considered together with ICA testing. The capture antibody on the Test line (T line) and the control antibody (goat-anti-mouse Ig G, Beyotime Biotech, Shanghai, China) on the Control line (C line) (Fig. 1) measured 2.0 mg/ml, and the labeled antibody exceeded 40 μg/ml. A 0.3 μg/ml of the S domain of VP1 was used. The final selection was based on a combination of the color effect of both C and T lines.

### HuNoVs in clinical samples (including suspected HuNoVs)

A total of 122 fecal specimens were collected and tested. Five HuNoVs clinical samples [57,404 (GI.1), 3010 (GI.1), 1704 (GII.4), 1717 (GII.4), 1028 (GII.4)] were provided by Dr. Ningbo Liao (Zhejiang Provincial Center for Disease Control and Prevention (CDC)). In total 117 clinical diarrheal samples were provided by the Affiliated Hospital of Guangzhou Medical University (3), the Affiliated Hospital of Sun Yat-sen University (7), Chinese CDC (39), and Anhui Provincial CDC (68). The samples were tested by both RT-qPCR and the developed ICA. Clinical samples provided by Zhejiang Provincial and Chinese CDC were obtained from patients with acute gastroenteritis in 2018. The samples from Anhui provincial CDC and Affiliated Hospital of Guangzhou Medical University were collected in 2017. The samples from the Affiliated Hospital of Sun Yat-sen University were obtained between 2015 and 2017.

### Detection of HuNoVs by RT-qPCR and calculation of viral genomic copies

Real-time RT-PCR was performed using a commercial one-step RT-qPCR kit (Sangon Biotech, China), consisting of 12.5 μl 2 × one-step RT-qPCR Master Mix (with SYBR Green), 0.65 μl RT enzyme Mix, and 0.4 μl of each primer (0.16 μmol/l) (See Table S7, Additional file 6) [51]. The RNA template (2.0 μl) and RNase free ddH_2_O were added to make a total volume of 25.0 μl. The amplification reactions were as follows: reverse transcription at 50 °C for 30 min; heat-denaturation at 95 °C for 3 min; 40 cycles with denaturation at 95 °C for 10 s, annealing and extension at 60 °C for 30 s. Fluorescence signals were collected at the end of each extension step. The highest dilution from real-time RT-PCR was used to generate a positive cycle threshold (C_*t*_) signal, which was one real-time RT-PCR unit (RT-qPCRU) [14]. Linear standard curves of C_*t*_ values versus log_10_ viral genomic copies were generated from a continuous 10-fold dilution (See Fig. S4, Additional file 7). Amplified DNA fragments were sequenced on an ABI 3730XL (Personalbio, China). Automated genotypes were analyzed using a Norovirus Typing Tool Version 2.0 (www.rivm.nl/mpf/norovirus/typingtool).

### Exposure of the S domain of VP1 from viral capsid in clinical samples

Pretreatment included physical or chemical methods. Heat-denaturation was used for physical treatment. Briefly, 10.0% (w/v) fecal sample was diluted with PBS, placed in a water bath at a specific temperature (60 °C, 70 °C, 80 °C, 90 °C, 100 °C) for 1 to 5 min at an interval of 1 min. A reducing agent (DTT) (Thermo Fisher, China) and alkaline conditions were used for chemical treatment. The pH in PBS was adjusted from 6.0 to 10.0. The fecal samples were dissolved to 10.0% with PBS buffers (pH 6.0, 7.0, 8.0, 9.0 or 10.0) at room temperature (25 °C) for 5, 10, 15, 20, 25 and 30 min. DTT was added to the homogenized solution of the clinical sample at a final concentration of 1.0%. Sandwich ELISA was used to detect HuNoVs, as previously described [52].

### Performing an ICA test

The procedure used to prepare ICA strips is described in Additional file 8. The sample preparation process was as follows: 10.0% (w/v) homogenized solution of stool slurries was prepared with PBS (pH 9.0) (10,000×g, 10 min). DTT was added to a final concentration of 1.0% and incubated at 25 °C for 10 min. 50 μl of the mixtures were added to the sample pad. In the presence of adequate amounts of HuNoVs antigens, the binding of the gold-labeled antigen complex occurred at both the T and C lines. The presence of C line confirmed that the test was valid.

### Limit of detection (LOD) of ICA for the purified S domain of VP1 in clinical samples

The purified S domain of the VP1 was used at concentrations of 22.4 ng/ml, 11.2 ng/ml, 5.6 ng/ml, 2.8 ng/ml, 1.4 ng/ml and 0.7 ng/ml. PBS was used as blank control. Two HuNoVs positive samples (57,404 GI.1 and 1717 GII.4) were two-fold diluted with 1.6 × 10^5^ to 5 × 10^6^ gc/g (GI) and 1.1 × 10^5^ to 3.5 × 10^6^ gc/g (GII). All experiments were performed in triplicate to confirm the reproducibility of the results.

### Evaluating ICA specificity

To determine the specificity of antibodies in the colloidal gold test, 4 Rotavirus, 3 Sapovirus, 2 Astrovirus and 4 Adenovirus (stool sample provided by Zhejiang CDC, the affiliated Hospital of Guangzhou Medical University and the affiliated Hospital of Sun Yat-sen University) and 3 *Salmonella* (ATCC 14028, CMCC 50115 and CICC 21482) were tested. The copies of Rotavirus, Sapovirus, Astrovirus, and Adenovirus RNA were more than 10^7^ gc/g feces.

### Stability test

Thermal acceleration tests were used to determine the stability of the ICA strips, at 60 °C [53]. The activity of the antibodies on each assay was determined using the lowest detectable S domain of the VP1 concentration (1.4 ng/ml). The identical strips were tested at appropriate intervals (1, 2, 3, 7, 14, 21, and 28 days). The shelf-life was estimated to be at room temperature.

### Statistical analysis

One-way ANOVA was used for data analysis. SPSSAU, an online data analysis tool, was used to perform all the statistical analyses (www.spssau.com).

## Supplementary Information

**Additional file 1: Figure S1.** A sandwich ELISA for detecting HuNoVs at diverse processing temperature and time. (∗∗ 0.01 < *p* < 0.05; ∗∗∗ *p* < 0.01). **Figure S2.** A sandwich ELISA analysis of HuNoVs after treatment of pH at different time. (∗∗ 0.01 < *p* < 0.05; ∗∗∗ *p* < 0.01).

**Additional file 2: Table S1.** The C_*t*_ data of five stool samples used in the LOD test. **Table S2.** The Ct data and viral genomic copies of 15 stool samples not detected by ICA.

**Additional file 3: Table S3.** Commercial kits for sensitivity, specificity and agreement comparison.

**Additional file 4: Table S4.** Optimal BSA concentration for blocking. **Table S5.** Selection of conjugate pad and dilution multiple of gold-labeled antibody. **Table S6.** (A) Selection of the Nitrocellulose Membrane. (B) Optimization of the capture antibody and control antibody concentration. (C) The optimal concentration of sucrose (w/v) for the immobilized antibody on the NC membrane.

**Additional file 5: Figure S3.** Selection of optimal conditions for gold-labeled antibody complex.

**Additional file 6: Table S7.** Primers used in RT-qPCR.

**Additional file 7: Figure S4.** Standard curves for calculation of genomic copies of GI and GII HuNoVs.

**Additional file 8.** Preparation of the sample pad and others and assembling of the ICA.

## Data Availability

The datasets supporting the conclusions of this article are included within the article and its additional files. The datasets used and/or analyzed during the current study are available from the corresponding author on reasonable request.

## Abbreviations

CI: Confidence interval
ATCC: American type culture collection
CMCC: National Center for Medical Culture collections
CICC: China Center of Industrial Culture Collection
ELISA: Enzyme-linked immunosorbent assay
DTT: Dithiothreitol
