# Supplementary material for: Broad-range and effective detection of human noroviruses by colloidal gold immunochromatographic assay based on the shell domain of the major capsid protein
Source: BMC Microbiol. 2021 Jan 11;21:22. doi: 10.1186/s12866-020-02084-z (PMC7798207; doi:10.1186/s12866-020-02084-z)

Additional file 1:

Fig. S1 A sandwich ELISA for detecting HuNoVs at diverse processing temperature and time. (∗∗ 0.01 <*p*< 0.05; ∗∗∗*p*< 0.01)


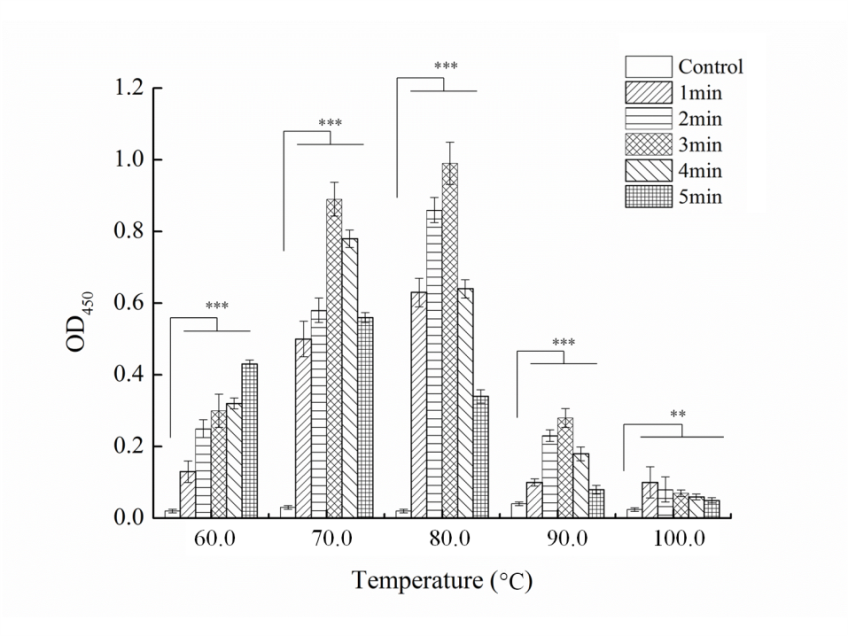


Additional file 1:

Fig. S2 A sandwich ELISA analysis of HuNoVs after treatment of pH at different time. (∗∗ 0.01 <*p*< 0.05; ∗∗∗*p*< 0.01)


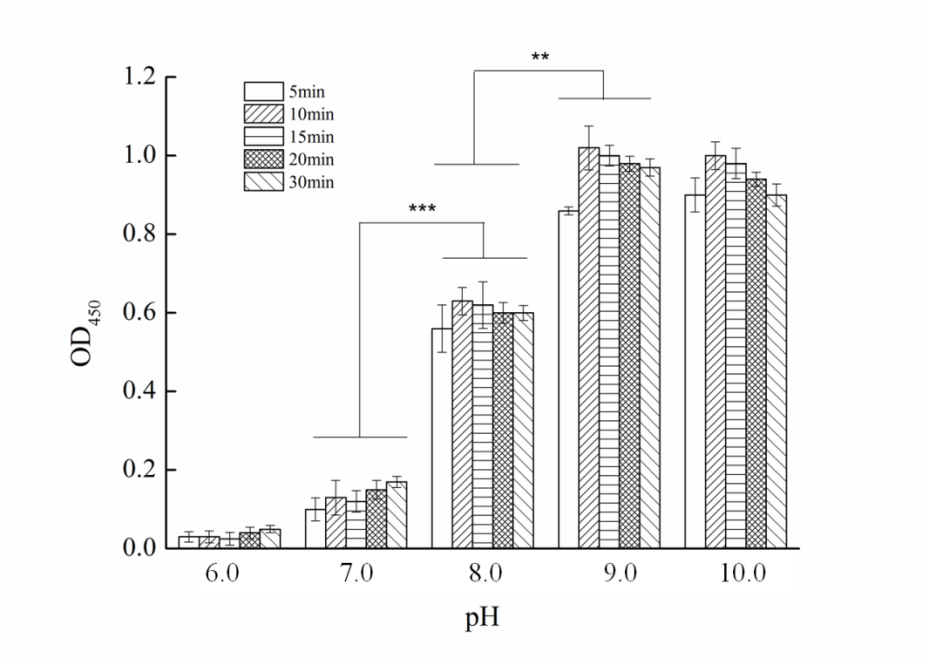

Supplement: Supplementary file 1 — Additional file 1: Figure S1. A sandwich ELISA for detecting HuNoVs at diverse processing temperature and time. (∗∗ 0.01 < p < 0.05; ∗∗∗ p < 0.01). Figure S2. A sandwich ELISA analysis of HuNoVs after treatment of pH at different time. (∗∗ 0.01 < p < 0.05; ∗∗∗ p < 0.01). [file 12866_2020_2084_MOESM1_ESM.docx]
