# Supplementary material for: Broad-range and effective detection of human noroviruses by colloidal gold immunochromatographic assay based on the shell domain of the major capsid protein
Source: BMC Microbiol. 2021 Jan 11;21:22. doi: 10.1186/s12866-020-02084-z (PMC7798207; doi:10.1186/s12866-020-02084-z)
Supplement: Supplementary file 2 — Additional file 2: Table S1. The Ct data of five stool samples used in the LOD test. Table S2. The Ct data and viral genomic copies of 15 stool samples not detected by ICA. [file 12866_2020_2084_MOESM2_ESM.docx]

**Additional file 2:**

**TableS1.** The C*_t_* data of five stool samples used in the LOD test

|  | 57404 (GI.1) | 3010 (GI.1) | 1704 (GII.4) | 1717 (GII.4) | 1028 (GII.4) |
| --- | --- | --- | --- | --- | --- |
| C*_t_* | 18.45 | 32.95 | 24.12 | 19.30 | 28.91 |
| PCRU | 619650 | 32 | 13125 | 347686 | 506 |
| Copies | 6.20E+10 | 3.24E+06 | 1.31E+09 | 3.48E+10 | 5.06E+07 |

**Additional file 2: Table S2.** The C*_t_* data and viral genomic copies of 15 stool samplesnot detected by ICA

|  | HuNoVs Genotypes | C*_t_* | Copies/g |
| --- | --- | --- | --- |
| 1 | GI.6 | 18.06 | 8.23E+05 |
| 2 | GII.3 | 20.27 | 1.67E+05 |
| 3 | GII.6 | 19.63 | 2.71E+05 |
| 4 | GII.4 | 21.31 | 7.56E+04 |
| 5 | GII.4 | 22.35 | 3.45E+04 |
| 6 | GII.4 | 21.04 | 9.28E+04 |
| 7 | GII.4 | 19.11 | 4.02E+05 |
| 8 | GII.4 | 22.83 | 2.39E+04 |
| 9 | GII.4 | 21.96 | 4.63E+04 |
| 10 | GII.4 | 20.86 | 1.07E+05 |
| 11 | GII.4 | 22.04 | 4.35E+04 |
| 12 | GII.4 | 23.10 | 1.96E+04 |
| 13 | GII.4 | 22.55 | 2.97E+04 |
| 14 | GII.4 | 20.07 | 1.94E+05 |
| 15 | GII.4 | 19.03 | 4.26E+05 |

**Note:** The LOD of ICA: GI: 1.2×10^6^copies/g; GII: 4.4×10^5^copies/g.
