# Supplementary material for: Broad-range and effective detection of human noroviruses by colloidal gold immunochromatographic assay based on the shell domain of the major capsid protein
Source: BMC Microbiol. 2021 Jan 11;21:22. doi: 10.1186/s12866-020-02084-z (PMC7798207; doi:10.1186/s12866-020-02084-z)
Supplement: Supplementary file 3 — Additional file 3: Table S3. Commercial kits for sensitivity, specificity and agreement comparison. [file 12866_2020_2084_MOESM3_ESM.docx]

**Additional file 3:**

**Table S3.** Commercial kits for sensitivity, specificity and agreement comparison

|  | Sensitivity | Specificity | Agreement | Reference |
| --- | --- | --- | --- | --- |
| ICA kit in this study | (80/95) 84.2 % | (27/27) 100.0 % | (107/122) 87.7 % |  |
| IP-NoV kit | (14/19) 73.7 % | (10/10) 100.0 % | (24/19) 95.2 % | [1] |
|  | (24/33) 72.7 % | (20/22) 90.9 % | (44/55) 80.0 % | [2] |
|  | (46/62) 74.2 % | (399/401) 99.5 % | (445/463) 96.1 % | [3] |
|  | (90/114) 78.9 % | (375/389) 96.4 % | (465/503) 92.4 % | [4] |
| QuickEx-Norovirus | (24/44) 54.5 % | (40/43) 93.0 % | (64/87) 73.6 % | [5] |
| RIDA® QUICK | (66/80) 82.5 % | (100/100) 100.0 % | (166/180) 92.2 % | [6] |
|  | (66/96) 68.8 % | (59/60) 98.3 % | (125/156) 80.1 % | [7] |
| RIDA® QUICK  (N1402) | (87/100) 87.0 % | (97/100) 97.0 % | (184/200) 92.0 % | [8] |
|  | (91/125) 72.8 % | (640/643) 99.5 % | (731/768) 95.2 % | [9] |
| ImmunoSearch NV kit | (46/61) 75.4 % | (14/14) 100.0 % | (60/75) 80.0 % | [10] |
| NOROTOP+ | (76/148) 51.4 % | （25/25) 100.0 % | (101/173) 58.4 % | [11] |
| SD Bioline Norovirus | (52/68) 76.5 % | (342/343) 99.7 % | (394/411) 95.9 % | [12] |
|  | (83/92) 90.2 % | (126/126) 100.0 % | (209/218) 95.9 % | [13] |

Reference

1. Nguyen TA, Khamrin P, Takanashi S, Le Hoang P, Pham le D, Hoang KT, Satou K, Masuoka Y, Okitsu S, Ushijima H. Evaluation of immunochromatography tests for detection of rotavirus and norovirus among Vietnamese children with acute gastroenteritis and the emergence of a novel norovirus GII.4 variant. J Trop Pediatr. 2007;53(4):264-9. doi: 10.1093/tropej/fmm021
2. Okame M, Yan H, Akihara S, Okitsu S, Tani H, Matsuura Y, Ushijima H.Evaluation of a newly developed immunochromatographic method for detection ofnorovirus. KansenshogakuZasshi. 2003;77(8):637-9.doi: 10.11150/kansenshogakuzasshi1970.77.637
3. Thongprachum A, Khamrin P, Chaimongkol N, Malasao R, Okitsu S, Mizuguchi M,Maneekarn N, Ushijima H. Evaluation of an immunochromatography method for rapiddetection of noroviruses in clinical specimens in Thailand. J Med Virol. 2010;82(12):2106-9.doi: 10.1002/jmv.21916
4. Khamrin P, Nguyen TA, Phan TG, Satou K, Masuoka Y, Okitsu S, ManeekarnN,Nishio O, Ushijima H. Evaluation of immunochromatography and commercialenzyme-linked immunosorbent assay for rapid detection of norovirus antigen instool samples. J Virol Methods. 2008;147(2):360-3.doi: 10.1016/j.jviromet.2007.09.007
5. Mutoh K, Hakamata A, Yagi H, Kurokawa K, Miki N, Kurita I. Evaluation of newcommercial immunochromatography kit for norovirus in feces. PediatrInt. 2009;51(1):164-6. doi: 10.1111/j.1442-200X.2008.02788.x.
6. Bruggink LD, Witlox KJ, Sameer R, Catton MG, Marshall JA. Evaluation of theRIDA(®)QUICK immunochromatographic norovirus detection assay using specimens fromAustralian gastroenteritis incidents. J Virol Methods. 2011;173(1):121-6.doi: 10.1016/j.jviromet.2011.01.017.
7. Kirby A, Gurgel RQ, Dove W, Vieira SC, Cunliffe NA, Cuevas LE. An evaluationof the RIDASCREEN and IDEIA enzyme immunoassays and the RIDAQUICKimmunochromatographic test for the detection of norovirus in faecal specimens. J Clin Virol. 2010;49(4):254-7. doi: 10.1016/j.jcv.2010.08.004.
8. Bruggink LD, Dunbar NL, Marshall JA. Evaluation of the updated RIDA®QUICK (Version N1402) immunochromatographic assay for the detection of norovirus in clinical specimens. J Virol Methods. 2015;223:82-7. doi: 10.1016/j.jviromet.2015.07.019.
9. Jonckheere S, Botteldoorn N, Vandecandelaere P, Frans J, Laffut W, Coppens G, Vankeerberghen A, De Beenhouwer H. Multicenter evaluation of the revised RIDA®QUICK test (N1402) for rapid detection of norovirus in a diagnostic laboratorysetting. Diagn Microbiol Infect Dis. 2017;88(1):31-35. doi:10.1016/j.diagmicrobio.2017.02.006.
10. Khamrin P, Takanashi S, Chan-It W, Kobayashi M, Nishimura S, Katsumata N, Okitsu S, Maneekarn N, Nishio O, Ushijima H. Immunochromatography test for rapid detection of norovirus in fecal specimens. J Virol Methods. 2009;157(2):219-22. doi: 10.1016/j.jviromet.2008.12.012
11. Ambert-Balay K, Pothier P. Evaluation of 4 immunochromatographic tests for rapid detection of norovirus in faecal samples. J Clin Virol. 2013;56(3):194-8. doi: 10.1016/j.jcv.2012.11.001
12. Park KS, Baek KA, Kim DU, Kwon KS, Bing SH, Park JS, Nam HS, Lee SH, Choi YJ. Evaluation of a new immunochromatographic assay kit for the rapid detection of norovirus in fecal specimens. Ann Lab Med. 2012;32(1):79-81. doi: 10.3343/alm.2012.32.1.79
13. Kim HS, Hyun J, Kim JS, Song W, Kang HJ, Lee KM. Evaluation of the SD Bioline Norovirus rapid immunochromatography test using fecal specimens from Korean gastroenteritis patients. J Virol Methods. 2012;186(1-2):94-8. doi: 10.1016/j.jviromet.2012.08.014
