# Supplementary material for: Broad-range and effective detection of human noroviruses by colloidal gold immunochromatographic assay based on the shell domain of the major capsid protein
Source: BMC Microbiol. 2021 Jan 11;21:22. doi: 10.1186/s12866-020-02084-z (PMC7798207; doi:10.1186/s12866-020-02084-z)
Supplement: Supplementary file 4 — Additional file 4: Table S4. Optimal BSA concentration for blocking. Table S5. Selection of conjugate pad and dilution multiple of gold-labeled antibody. Table S6. (A) Selection of the Nitrocellulose Membrane. (B) Optimization of the capture antibody and control antibody concentration. (C) The optimal concentration of sucrose (w/v) for the immobilized antibody on the NC membrane. [file 12866_2020_2084_MOESM4_ESM.docx]

**Additional file 4:**

**Table S4.** Optimal BSA concentration for blocking

| BSA final concentration (%) | 3.0 | 2.5 | 2.0 | 1.5 | 1.0 | 0.5 |
| --- | --- | --- | --- | --- | --- | --- |
| Colloidal gold state | - | - | - | + | + | + |
| Background color effect | ND | ND | ND | * | ** | *** |
|  |  |  |  |  |  |  |
| ’**-:** coagulation **+:** normality ND**:** not detected * **:**color level | | | | | | |

**Additional file 4:**

**Table S5.** Selection of conjugate pad and dilution multiple of gold labeled antibody

| Fiber | 1:1 | 1:2 | 1:4 | 1:8 |
| --- | --- | --- | --- | --- |
| Glass fiber (Ahlstrom 8964, Whatman USA) | * | ** | *** | *** |
| Polyester fiber (fusion 3, Whatman, USA) | * | * | ** | ** |

* Release effect

**Note:** Two conjugate pads were selected for comparison: the glass fiber Ahlstrom 8964 (Whatman, USA) and the polyester material fusion 3 (Whatman, USA). In addition, colloidal gold-antibody mixture was diluted with dissolution buffer in gradient of 1:1, 1:2,1:4 and 1:8 to find the best spraying amount. S protein (0.3 μg/ml) was used to test the most suitable amount of spray. Glass fiber was finally selected for conjugate pad with a spraying amount of 1:4.

**Additional file 4:**

**Table S6. (A)** Selection of the Nitrocellulose Membrane. (**B)** Optimization of the capture antibody and control antibody concentration. (**C)** Optimal concentrationof sucrose (w/v) for the immobilized antibody on NC membrane.

**(A)**Selection of the Nitrocellulose Membrane

|  | Flow time (s) | Fixation effect | Background color |
| --- | --- | --- | --- |
| CN 140 (Sartorius, Germany) | 212±2 | ** | * |
| CN 95 (Sartorius, Germany) | 143±4 | * | * |
| M135 (Millipore, USA) | 203±3 | * | * |
| M180 (Millipore, USA) | 256±2 | ** | ** |

**Note:**Quantity of *denoted the color intensity

**(B)**Optimization of the capture antibody and control antibody concentration

| Control line IgG(mg/ml)  Test line J5D (mg/ml) | 1.0 | 1.2 | 1.4 | 1.6 | 1.8 | 2.0 |
| --- | --- | --- | --- | --- | --- | --- |
| 0.5 | *＊ | *＊ | *＊ | *＊＊ | *＊＊ | *＊＊ |
| 1.0 | *＊ | *＊ | *＊ | *＊ | *＊＊ | *＊＊ |
| 1.5 | **＊ | **＊ | **＊＊ | **＊＊ | **＊＊＊ | **＊＊＊ |
| 2.0 | ***＊ | ***＊＊ | ***＊＊ | ***＊＊＊ | ***＊＊＊ | ***＊＊＊ |
| 2.5 | ***＊ | ***＊ | ***＊ | ***＊＊ | ***＊＊ | ***＊＊ |
| 3.0 | *** | *** | ***＊ | **＊ | ***＊ | ***＊＊ |
| *Test line ＊Control lineQuantity of *and＊denoted the color intensity. | | | | | | |

**(C)**Optimal concentration of sucrose (w/v) for the immobilized antibody on NC membrane.

| Sucrose Concentration (%) | 0.0 | 1.0 | 2.0 | 3.0 | 4.0 | 5.0 | 6.0 |
| --- | --- | --- | --- | --- | --- | --- | --- |
| Immobilized Antibody | ***** | ***** | ****** | ******* | ****** | ****** | ****** |

**Note:**Quantity of *denoted the color intensity

Four NC membranes were selected as candidates (Table S6): CN 140 (Sartorius, Germany), CN 95 (Sartorius, Germany), M135 (Millipore, USA), M180 (Millipore, USA). CN140 was finally selected with the merits of slower flow rate, higher sensitivity and less background color (Table S6 A).Optimal concentration of capture antibody used in the Test line (J5E) and the Control line (goat-anti-mouse Ig G (Beyotime Biotech, Shanghai, China) were tested with a range of 0.5 to 3.0 mg/ml and 1.0 to 2.0 mg/ml, respectively (Table S6 B). Finally, 2.0 mg/ml J5E and 1.6 mg/ml goat-anti-mouse Ig G was selected to draw T and C lines by using the XYZ3010 instrument (Jiening Biotechnology, Shanghai). The distance between the two lines was 5 mm. To get better fixation and protection effect, 0, 1.0 %, 2.0 %, 3.0 %, 4.0 %, 5.0 % and 6.0 % (w/v) of sucrose were evaluated in the diluted MAb and IgG for immobilized antibodies on NC membrane. The results indicated that 3.0% sucrose was the best (Table S6 C).
