# Supplementary material for: Broad-range and effective detection of human noroviruses by colloidal gold immunochromatographic assay based on the shell domain of the major capsid protein
Source: BMC Microbiol. 2021 Jan 11;21:22. doi: 10.1186/s12866-020-02084-z (PMC7798207; doi:10.1186/s12866-020-02084-z)
Supplement: Supplementary file 5 — Additional file 5: Figure S3. Selection of optimal conditions for gold-labeled antibody complex. [file 12866_2020_2084_MOESM5_ESM.docx]

**Additional file 5:**

**Preparation of the colloidal gold labeled MAbs**

Colloidal gold particles with a mean particle diameter of 25.0 nm were produced under the following procedures. One hundred ml 0.01% (w/v) chloroauric acid (HAuCl_4_) (Aladdin, Shanghai, China) was boiled thoroughly for 3min. Then 2.0 ml 1.0 %(w/v) sodium citrate (Aladdin, Shanghai, China) was added quickly into the solution on a magnetic stirring apparatus over 30 min. The color changed gradually from yellow to black–blue and finally brilliant red. After stirring for a few minutes at low speed, the colloidal gold suspension was rested to cool down and stored in the dark case at roomtemperature. Total volume was made up to the original volume (100.0 ml) by adding ultrapure water. To measure the size and size distribution of these gold nanoparticles, the colloidal gold solution was scanned under a transmission electron microscopy (Tecnai G2 spirit Biotwin, USA) at 120 KV. The OD value of the colloidal gold solution was measured at 400-680 nm using an ultraviolet spectrophotometer (Tecan Sunrise, Switzerland).

The optimal pH, dose and concentration of BSA for conjugation of gold colloids and H9E monoclonal antibodies were evaluated (Fig. S3and Table S5). Briefly, 200.0μl of H9E was incubated with 0.5 ml of colloidal gold (pH 9.0) for 30 min at room temperature with stirring gently. Then, 50.0 µl bovine serum albumin (BSA, Amresco, United States) of different final concentration were dripped into the colloidal gold as the blocking buffer to stabilize the gold-labeled antibody. After incubated for 15 min, the colloidal gold-antibody complex was collected in pellet and unmarked antibodies were remained in supernatant by a centrifugation at 8000 ×g at 4 °C for 20 min. Centrifugal process should be avoided the presence of black massive deposits on the wall of the tube. Then, the conjugated colloidal gold-antibody was finally resuspended to 50.0 μl dissolution buffer pH 9.0 PBS containing 10.0 % w/v sucrose (Sangon Biotech, Shanghai, China), 0.2% (w/v) PVA-205 (Aladdin, Shanghai, China), 0.2% (v/v) Tween-20 (Aladdin, Shanghai, China) and BSA (3.0%, 2.5%, 2.0%, 1.5%, 1.0% and 0.5%, w/v) respectively. The conjugation was confirmed by UV-vis spectroscopy using the same method as unlabeled gold particles. Ultimately, 50.0 μl of colloidal gold-antibody mixture was dispensed evenly on the 0.5 cm×2.5 cm conjugated pad and was dried for 3 h at room temperature.

**(A)**


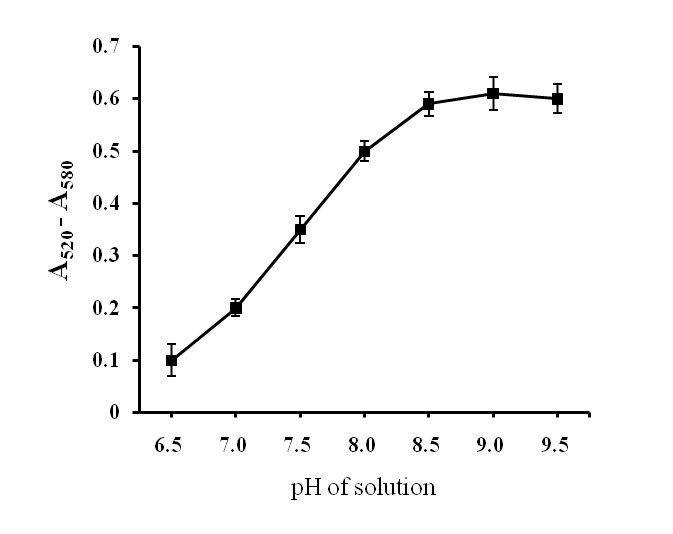


**(B)**


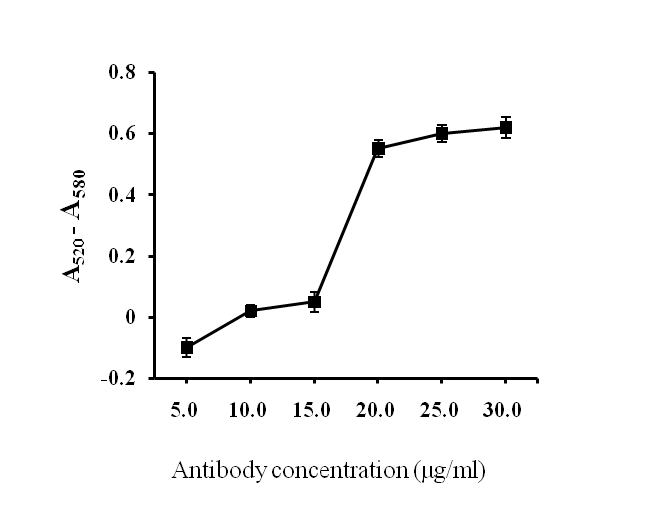


**Additional file 5: Fig. S3** Selection of optimal conditions for gold labeled antibody complex. (**A)**Evaluation of the optimal pH. Five hundred μl of colloidal gold solution was added to eight sterile EP tubes, respectively. The pH values were modified to 6.5 to 9.5 by adding 0.2 M potassium carbonate (K_2_CO_3_) or0.1M hydrochloric acid (HCl). Two hundred µl of H9E (50.0 μg/ml) was added into each tube with gentle stirring for 30 min, and then 50.0 µl NaCl (10.0 % w/v) was added to the above tubes. The mixture was permitted to stand for 2 h at room temperature. The OD_520_ and OD_580_ values were measured in accordance with an ultraviolet spectrophotometer (Eppendorf Bio Spectrometer, Germany).The optimal pH and labeled antibody concentration conditions can be determined by measuring A_520_-A_580_, the differential absorbance.**(B)** Dose for conjugation of 1.0 ml gold colloids and H9E monoclonal antibodies (μg). Various amounts of H9E antibodies were mixed with 500.0µl of colloid gold at pH 9.0 and 1.0 % BSA.
