# Supplementary material for: Broad-range and effective detection of human noroviruses by colloidal gold immunochromatographic assay based on the shell domain of the major capsid protein
Source: BMC Microbiol. 2021 Jan 11;21:22. doi: 10.1186/s12866-020-02084-z (PMC7798207; doi:10.1186/s12866-020-02084-z)
Supplement: Supplementary file 6 — Additional file 6: Table S7. Primers used in RT-qPCR. [file 12866_2020_2084_MOESM6_ESM.docx]

**Additional file 6:**

**Table S7.** Primers used in RT-qPCR

| HuNoVs | Primers | | | |
| --- | --- | --- | --- | --- |
| GI | COG1F | 5’- | CGY TGG ATG CGI TTY CAT GA | -3’ |
|  | COG1R |  | CTT AGA CGC CAT CATCAT TYA C |  |
| GII | COG2F |  | CAR GAR BCN ATG TTY AGR TGG ATG AG |  |
|  | COG2R |  | TCG ACG CCA TCT TCA TTC ACA |  |

**Note:** COG1F and COG2F were forward primers; COG1R and COG2R were reverse primers. Y = C or T; I = inosine; R = A or G; B = C, G or T; N = A, C, G, or T; Primers were selected against a highly conserved region, ORF1-ORF2 junction.
