# Supplementary material for: Broad-range and effective detection of human noroviruses by colloidal gold immunochromatographic assay based on the shell domain of the major capsid protein
Source: BMC Microbiol. 2021 Jan 11;21:22. doi: 10.1186/s12866-020-02084-z (PMC7798207; doi:10.1186/s12866-020-02084-z)
Supplement: Supplementary file 7 — Additional file 7: Figure S4. Standard curves for calculation of genomic copies of GI and GII HuNoVs. [file 12866_2020_2084_MOESM7_ESM.docx]

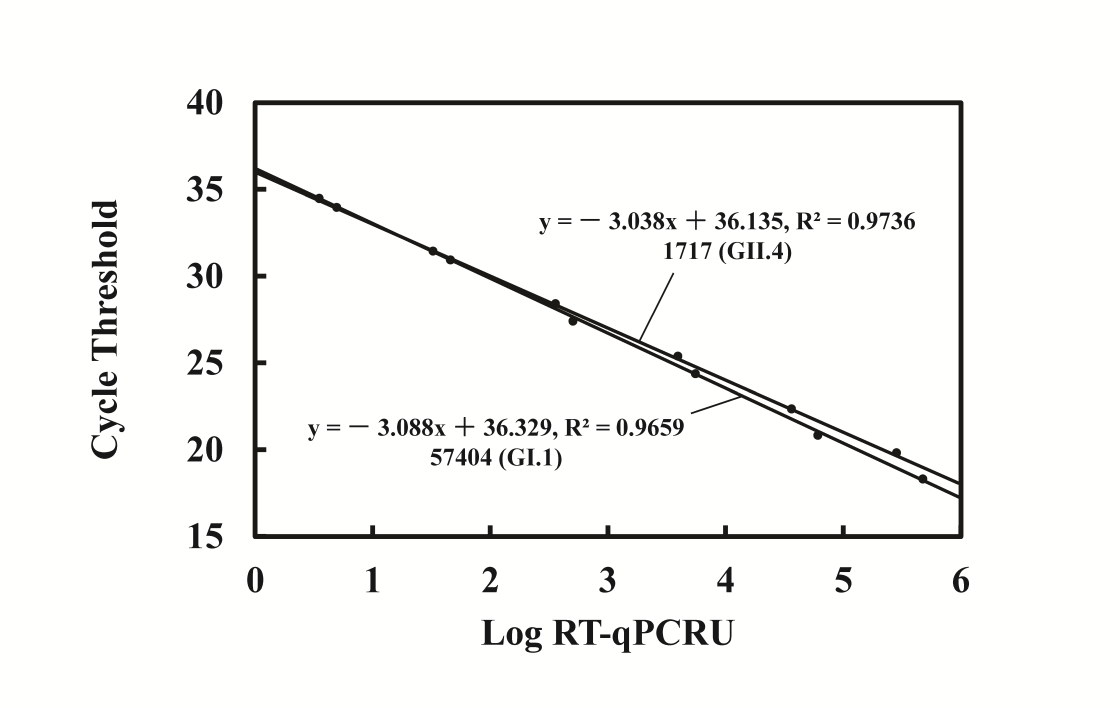


**Additional file 7: Fig. S4** Standard curves for calculation of genomic copies of GI and GII HuNoVs. Preliminary tests showed that sample 57404 (GI) and 1717 (GII) had the highest viral titer in feces. These two samples were used to generate the standard curve for GI and GII. The linear regression equation of standard curve for GI was y = − 3.088x + 36.329 (x, log RT-qPCRU; y, C*_t_*) and the correlation coefficient (R^2^) was 0.9659; for GII was y= − 3.038x + 36.135 (x, log RT-qPCRU; y, C*_t_*) and the R^2^ was 0.9736.
