# Supplementary material for: Broad-range and effective detection of human noroviruses by colloidal gold immunochromatographic assay based on the shell domain of the major capsid protein
Source: BMC Microbiol. 2021 Jan 11;21:22. doi: 10.1186/s12866-020-02084-z (PMC7798207; doi:10.1186/s12866-020-02084-z)
Supplement: Supplementary file 8 — Additional file 8. Preparation of the sample pad and others and assembling of the ICA. [file 12866_2020_2084_MOESM8_ESM.docx]

**Additional file 8:**

**Preparation of the sample pad and others and assembling of the ICA**

The sample pad, absorbent pad and PVC board were provided by Jieyi Biotechnology Co., Ltd. (Shanghai, China). The sample pad was saturated with PBS solution (pH 9.0) containing 1.0% (w/v) BSA, 0.5% (w/v) PVA-205 and 0.5% (v/v) Tween-20 and dried before use. H5072 cellulose was used as the absorbent pad without treatment. The PVC board served as the bottom plate of the test strip.

The pre-treated conjugate pad, NC membrane, sample pad, and absorbent pad were assembled and immobilized on the PVC board as described in Fig. 3, overlapping 5 mm between each membrane. Then these strips were cut into 4 mm wide and stored in sealing bag with desiccant. The control line should always show a visible red line. The appearance of both two lines indicated a positive result.
